# Effectiveness of Thoracic Spine Manipulation for the Management of Neck Pain: A Systematic Umbrella Review with Risk of Bias and Methodological and Reporting Quality

**DOI:** 10.3390/healthcare14020240

**Published:** 2026-01-18

**Authors:** Michael Masaracchio, Kaitlin Kirker, Birendra Dewan, Stephen Caronia

**Affiliations:** Department of Physical Therapy, Long Island University, 1 University Plaza, Brooklyn, NY 11201, USA; kaitlin.kirker@liu.edu (K.K.); birendra.madidewan@liu.edu (B.D.); stephen.caronia@liu.edu (S.C.)

**Keywords:** manipulation, spinal, thoracic vertebrae, neck pain, reporting quality, methodological quality, risk of bias

## Abstract

Background/Objectives: The purpose of this umbrella review was to assess the risk of bias and the methodological and reporting quality of systematic reviews that evaluated the effects of thoracic spine manipulation (TSM) on individuals with mechanical neck pain. Methods: To be included, publications needed to be systematic reviews including studies with participants with neck pain >18 years old; at least two groups where the experimental intervention was TSM; assessed pain and/or function; and were published in English. Reviews limited to narrative, scoping, or retrospective studies, or those with cervical radiculopathy, were excluded. An electronic search was conducted in May 2025 using PubMed, CINAHL (EBSCO Host), and the Cochrane Library to identify relevant articles from inception to May 2025. Quality and risk of bias were assessed using A Measurement Tool to Assess Systematic Reviews 2 (AMSTAR 2), Preferred Reporting Items for Systematic Reviews and Meta-Analyses (PRISMA 2020), and Risk of Bias in Systematic Reviews (ROBIS). Findings were summarized narratively and graphically. Results: Seven reviews (27 unique studies; 1394 participants, aged 18–62 years) met the inclusion criteria. Some evidence supported TSM for short-term improvement in neck pain, but confidence in results was low to critically low based on the AMSTAR 2 results. Four reviews had a high overall risk of bias, and three had a low risk. Reporting compliance varied widely (0–100%). Conclusions: While all the included systematic reviews suggested that TSM is a viable short-term option for individuals with neck pain, the overall confidence in these results ranged from low to critically low, making it difficult to draw firm conclusions about the true benefit of TSM in clinical practice. Registered prospectively in PROSPERO (CRD420251034330).

## 1. Introduction

Neck pain is one of the most prevalent and costly medical conditions worldwide. According to the Global Burden of Disease, an estimated 203 million people worldwide experienced neck pain in 2020—a 77.3% increase since 1990 [1]. This number is projected to rise to 269 million by 2050, representing a further 32.5% increase from 2020 [1]. In the United States alone, neck pain ranked in the top 50 most costly medical conditions in 2019, with a total spending of $19.4 billion [2]. Accordingly, a multitude of interventions have been studied for their effectiveness in treating neck pain, including pharmacological, surgery, manual therapy, exercise, psychosocial/behavioral techniques, and multimodal approaches. Among these, manual therapy, which is often performed by a licensed physical therapist, has been repeatedly demonstrated to be effective in the management of neck pain [3,4,5,6,7,8,9,10]. Manual therapy is defined as passive, skilled movement applied by clinicians that directly or indirectly targets a variety of anatomical structures or systems, which is utilized with the intent to create beneficial changes in some aspect of the patient’s pain experience [11].

Spinal thrust manipulation is a specific manual therapy intervention defined as “a high velocity, low amplitude therapeutic movement within or at the end range of motion” [12]. While there still exists much debate on its mechanism of action, a wide breadth of neurophysiological mechanisms has been proposed as the processes by which spinal thrust manipulation functions. Neuromuscular responses [13], biomechanical effects [14], the descending pain modulatory system [15], presynaptic inhibition in the spinal cord [15], and sensorimotor integration [15] have all been studied and may each play a role in the effectiveness of spinal thrust manipulation in general. Innumerable studies in recent decades have investigated the use of spinal thrust manipulation across the stages of injury for the cervical, thoracic, and lumbar spine regions. The most recently updated Neck Pain Clinical Practice Guidelines of the American Physical Therapy Association (APTA) recommend spinal thrust manipulation for the management of individuals with neck pain with mobility deficits, with evidence levels of grade B and C [16]. Neck pain with mobility deficits, also considered non-specific mechanical neck pain, can be defined as pain between the occiput and cervicothoracic junction, that is exacerbated by static postures and movement without any specific cause [3].

The use of thoracic spine manipulation (TSM) for the management of neck pain has been increasingly studied in recent years, with several systematic reviews examining its effectiveness [17,18,19,20,21,22,23]. The foundational concept behind this approach is regional interdependence (RI), originally defined in the literature in 2007 as a “concept that seemingly unrelated impairments in a remote anatomical region may contribute to, or be associated with, the patient’s primary complaint” [24]. This paradigm essentially designated the effects of interventions directed toward remote body regions as being musculoskeletal/structural; however, the definition of RI has evolved over time [25]. Bialosky et al. posited that RI could also be driven by neurophysiological responses in addition to musculoskeletal [15]. Further, Sueki et al. [26] proposed that RI is also influenced by biopsychosocial and somatovisceral responses. Considering these potential mechanisms, several systematic reviews exploring the effect of TSM on neck pain have been conducted, comparing TSM to other treatment modalities [17,18,19,20,21,22,23]. Despite an abundance of evidence supporting the use of manual therapy in patients with spine pain for its effects on outcomes and cost-effectiveness [27,28,29,30], it has recently come under scrutiny for a lack of high-quality evidence and long-term effects [31,32,33,34,35]. A recent umbrella review assessed the quality of systematic reviews that evaluated the effectiveness of manual therapy in the management of different neck disorders [36]. However, that review focused on a variety of neck disorders and did not perform an appraisal of reporting quality or risk of bias in systematic reviews. While this study concluded high confidence in the results using the AMSTAR 2, known inconsistencies in reporting, methodological design, and risk of bias for TSM in the management of mechanical neck pain warrant the need for an additional focused umbrella review to guide future research design. Therefore, the purpose of this umbrella review is to assess the risk of bias and the methodological and reporting quality of systematic reviews that evaluated the effects of TSM on individuals with mechanical neck pain.

## 2. Materials and Methods

### 2.1. Protocol and Registration

This umbrella review followed the A Measurement Tool to Assess Systematic Reviews 2 (AMSTAR 2) criteria [37] and Preferred Reporting Items for Systematic Reviews and Meta-Analyses (PRISMA) 2020 guidelines [38]. It was registered prospectively in PROSPERO (CRD420251034330). PRISMA 2020 was selected exclusively because it represents the most current, comprehensive, and methodologically rigorous reporting guideline for systematic reviews. Although the Preferred Reporting Items for Overviews of Reviews (PRIOR) guidelines also outline items specific to umbrella reviews, PRISMA 2020 encompasses core reporting elements essential to umbrella reviews and has been widely adopted across health research disciplines. To ensure consistency, avoid redundancy, and maintain clarity in reporting, a single reporting standard was applied.

### 2.2. Protocol Changes

The authors initially planned to conduct a quantitative synthesis of primary randomized controlled trials (RCTs) through a meta-meta-analysis approach. However, methodological assessment revealed substantial overlap among the systematic reviews (corrected covered area > 15%) [39]. According to established guidance of Pieper et al., [39] such overlap violates the assumption of data independence required for meta-meta-analysis and inflates precision if pooled. Therefore, quantitative pooling was not pursued, and descriptive statistical and narrative summaries were performed instead.

### 2.3. Inclusion and Exclusion Criteria

To be included in this umbrella review, the study needed to be a systematic review that included studies (1) where the target population was participants with neck pain ≥ 18 years old; (2) with at least two groups where the experimental intervention was thoracic spine manipulation; (3) that assessed at least one outcome of pain or function; and (4) that were published in English. Literature, narrative, and scoping reviews were excluded. Systematic were also excluded if included studies had a retrospective design. For the purpose of this umbrella review, neck pain was operationally defined as non-specific mechanical neck pain or otherwise labeled neck pain with mobility deficits by the APTA’s clinical practice guidelines, without radicular symptoms. Therefore, systematic reviews involving patients with signs of cervical radiculopathy and symptoms consistent with nerve root involvement were also excluded.

### 2.4. Search Strategy and Study Selection

A search was conducted electronically in May 2025 by two independent authors (MM and KK) using CINAHL (EBSCO Host), PubMed, and the Cochrane Library to identify systematic reviews published from database inception to May 2025. The search strategy used truncation and Boolean operators ‘OR’ and ‘AND’ to combine MeSH Terms, CINAHL headings, subject headings, and keywords related to systematic reviews on thoracic spine manipulation and neck pain, such as thoracic spine manipulation, spinal manipulative therapy, high velocity low amplitude, mechanical neck pain, and cervicalgia. The full search strategy can be found in Table S1. Two authors (MM and KK) hand-searched the reference lists for additional studies that met inclusion criteria. Results of the literature search were uploaded to Endnote, where duplicates were removed, Two authors (KK and MM) independently screened titles and abstracts for eligibility. Full-text reviews were screened for inclusion and exclusion criteria by two authors (KK and MM). If consensus was not reached, a third reviewer (SC) provided a decision. When studies were excluded, the reason for exclusion was agreed upon by two authors (KK and MM) and recorded (Table S2).

### 2.5. Interventions

Manipulation has been defined as a high-velocity, small amplitude (grade V) therapeutic movement delivered at end range [11]. This umbrella review compared TSM to placebo TSM, cervical spine manipulation, modalities, exercises, and standard care. Exercise selection was highly variable but generally included some form of active range of motion and endurance training of the cervical and periscapular muscles [7,40,41,42]. Standard care was operationally defined as the combination of exercise and/or modalities [40,41,42,43,44,45].

### 2.6. Methodological Quality

The methodological quality of the included systematic reviews was evaluated using the AMSTAR 2 by two independent authors (KK and MM) [37]. Any discrepancies were resolved through consensus. The AMSTAR 2 includes 16 items that assess the overall quality of systematic reviews based on seven critical domains and nine non-critical domains [37]. On each of the criteria, “Yes” or “Partial Yes” was assigned depending on the level of adherence to the domain. “No” was assigned if the information available did not justify partial or full adherence to the criteria. For select items, “Not applicable” was assigned based on the study design of the included articles and if meta-analysis was not conducted. Calculation of a total score is not recommended as it may conceal deficiencies within specific domains [37]. However, the overall confidence in the results of the review can be interpreted by considering flaws in critical and non-critical domains. Shea et al. [37] proposed the following grading scheme.

1. High: No or one non-critical weakness. The systematic review provides an accurate and comprehensive summary of the results of the available studies that address the question of interest [37].
2. Moderate: More than one non-critical weakness. The systematic review may provide an accurate summary of the results of the available studies that were included in the review [37].
3. Low: One critical flaw with or without non-critical weaknesses. The review may not provide an accurate and comprehensive summary of the available studies that address the question of interest [37].
4. Critically low: More than one critical flaw with or without non-critical weaknesses. The review should not be relied on to provide an accurate and comprehensive summary of the available studies [37].

### 2.7. Reporting Quality

The reporting quality of the included systematic reviews was assessed by two independent authors (KK and MM) using the PRISMA 2020 guidelines [38], with discrepancies resolved through discussion. The PRISMA 2020 [38] guidelines are a 27-item checklist that assesses the completeness of reporting of systematic reviews and meta-analyses, which include 12 separate items for abstract reporting [38]. For each item, “Yes” was assigned if the information was satisfactorily stated in the review. “No” was assigned if the information was unclear, incomplete, or not reported. “Not applicable” was assigned if the criterion did not apply to the systematic review. Alternately, “Partial Yes” was considered if the information was reported in the review, but was located in the wrong section according to the PRISMA 2020 guidelines or when systematic reviews used the Physiotherapy Evidence Database (PEDro) scale [46] to assess methodological quality rather than a tool designed to assess risk of bias.

### 2.8. Risk of Bias

The risk of bias of the included systematic reviews was assessed independently by two authors (KK and MM) using the Risk of Bias in Systematic Reviews (ROBIS) Tool [47], with discrepancies resolved through discussion. The standardized tool involves a three-phase process: (1) assessing relevance, (2) identifying concerns with the review process across four domains (study eligibility criteria; identification and selection of studies; data collection and study appraisal; and synthesis and findings), and (3) judging the overall risk of bias.

### 2.9. Data Extraction

Data extraction was performed by two independent authors (KK and MM) for all studies using a structured pre-defined extraction form. Any discrepancy was resolved through discussion until consensus was reached. Data extracted included the primary author, year of publication, methodological design of included studies, sample size, participant demographics (i.e., sex, age, symptom duration), risk of bias/quality assessment tool used, patient population, experimental and comparison intervention details, follow-up period, and a summary of main conclusions (Table 1). Participants who received TSM were labeled the TSM group and participants who received other interventions were labeled the comparison group.

### 2.10. Data Synthesis

Although meta-meta-analysis would be consistent with best practice in an umbrella review, statistical evaluation of the meta-analyses in each included systematic review revealed that meta-meta-analysis would not be appropriate. The authors initially planned to conduct meta-meta-analysis using random-effect models in R using the “metafor” package. However, overlap of primary studies across reviews was quantified using the corrected covered area (CCA) method as described by Pieper et al. [39] to determine if quantitative pooling using meta-meta-analysis was appropriate. A low CCA (≤ 5%) indicates only a slight overlap of studies between reviews and supports the use of a random-effect meta-meta-analysis. Conversely, a very high CCA (>15%) reflects a substantial overlap of the primary studies across systematic reviews, which violates the assumption of independence and inflates the results of the overall effect estimate [39]. Consequently, rather than conducting meta-meta-analysis, the results of included systematic reviews regarding pain and function were synthesized narratively.

## 3. Results

### 3.1. Study Selection

**Figure 1:**
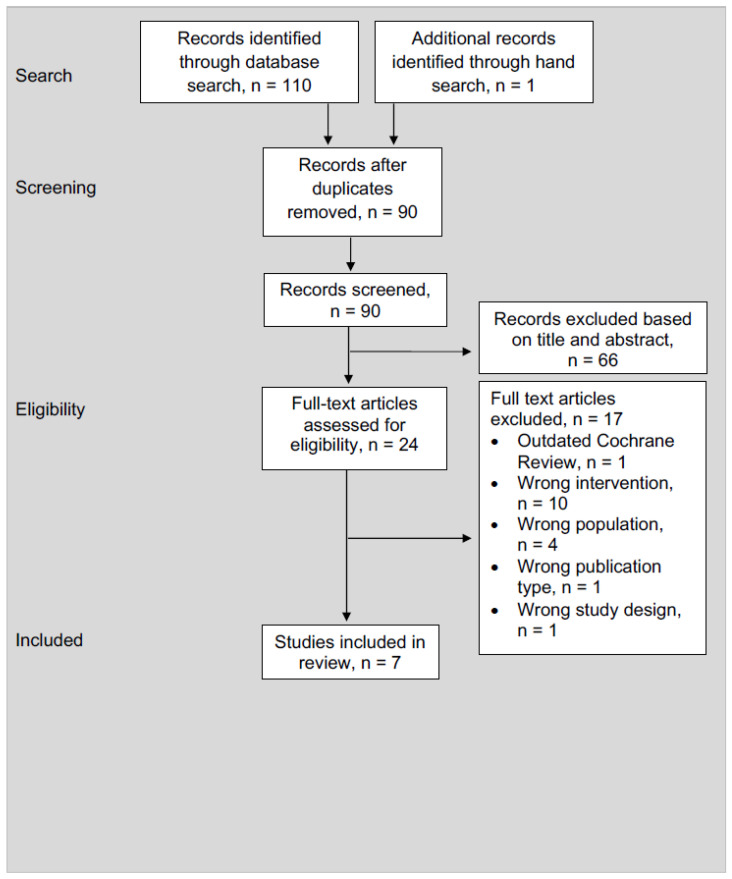
PRISMA flow diagram.

The search strategy identified 110 citations, with 91 titles and abstracts screened after duplicates were removed. A total of 25 full-text systematic reviews were assessed for eligibility and 18 were excluded (Table S2). Seven systematic reviews [17,18,19,20,21,22,23] with a total of 27 unique studies, were included (Figure 1).

### 3.2. Characteristics of Included Studies

Of the 27 unique included studies [3,4,7,9,40,41,42,43,44,45,48,49,50,51,52,53,54,55,56,57,58,59,60,61,62,63,64] across the seven systematic reviews, there were 23 RCTs, one secondary analysis [51], one quasi-experimental study [53], one prospective cohort study [48], and one case series [52]. All primary studies except for nine [41,48,49,50,52,53,55,56,64] were included in multiple systematic reviews. Across all 27 included studies, there were a total of 1394 participants. All seven systematic reviews evaluated the effectiveness of TSM on pain and function in patients with neck pain, with five systematic reviews [17,18,20,21,23] specifying mechanical neck pain and one described as “non-specific” [19]. Two systematic reviews focused on assessing participants with acute neck pain (≤3 months) [18,22], one examined only chronic cases [21], one did not specify symptom duration [23], and the remaining three included all durations [17,19,20]. The age of included participants ranged from 18 to 62 years old, and most of the systematic reviews did not synthesize patient demographics on gender distribution (Table 1).

Only three systematic reviews [20,21,22] conducted meta-analyses of patient outcome data, each employing markedly different designs in terms of comparison groups, outcome measures, and measurement instruments, which limited pooled synthesis of meta-analyses. Additionally, the degree of overlap in primary studies across the included systematic reviews was quantified using the CCA and pairwise Jaccard similarity index [39]. For the outcome of pain, the CCA was 23.7%, indicating a high degree of overlap among the included reviews. Pairwise Jaccard similarity showed moderate overlap (0.29) between Masaracchio et al. [20] and Tsegay et al. [21], moderate overlap (0.25) between Masaracchio et al. [20] and Walser et al. [22], and no overlap (0.0) between Tsegay et al. [21] and Walser et al. [22]. For the outcome of disability, the CCA was 25.0%, also reflecting high overlap. Pairwise Jaccard similarity indicated moderate overlap (0.22) between Masaracchio et al. [20] and Tsegay et al. [21], moderate overlap (0.33) between Masaracchio et al. [20] and Walser et al. [22], and no overlap (0.0) between Tsegay et al. [21] and Walser et al. [22]. Given that both outcomes demonstrated high overall overlap (CCA > 15%, which Pieper et al. [39] classify as “very high overlap”) and that pairwise Jaccard indices were non-negligible (>0.2) in multiple pairs, it was determined that conducting a meta-meta-analysis would have risked double-counting primary studies and inflating precision. Given the substantial overlap, the assumption of independent evidence is violated, warranting the decision to forego a meta-meta-analysis.

Three systematic reviews [20,21,23] conducted a Grading of Recommendations, Assessment, and Evaluation (GRADE) to determine the certainty of the evidence for the included primary studies in the associated systematic review, demonstrating very low to moderate quality. One additional systematic review [19] performed a best evidence qualitative synthesis based on the recommendations of van Peppen et al. [65], which found insufficient evidence that TSM is more effective than control interventions in reducing pain or disability.

### 3.3. Risk of Bias/Methodological Quality

Six systematic reviews assessed the methodological quality of the included studies using the PEDro scale [17,18,19,21,22,23] and one assessed risk of bias using the Cochrane Risk of Bias Tool [20] with variable ranges of quality and bias (Table 1).

**Figure 2:**
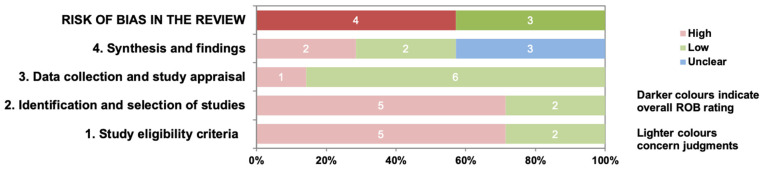
Risk of bias of included systematic reviews.

The risk of bias of the included systematic reviews was assessed using the ROBIS, determining high overall risk of bias in four systematic reviews [17,18,22,23], and low overall risk in three [19,20,21] (Figure 2). The domains demonstrating the highest concern for bias were Domains 1 and 2, related to the absence of review protocols and limitations of eligibility criteria and search strategies that may increase the risk of excluding relevant studies. Additional concerns from Domain 4 were related to the methods of data synthesis and the consideration of the risk of bias of the included studies when interpreting findings (Table S3).

**Figure 3:**
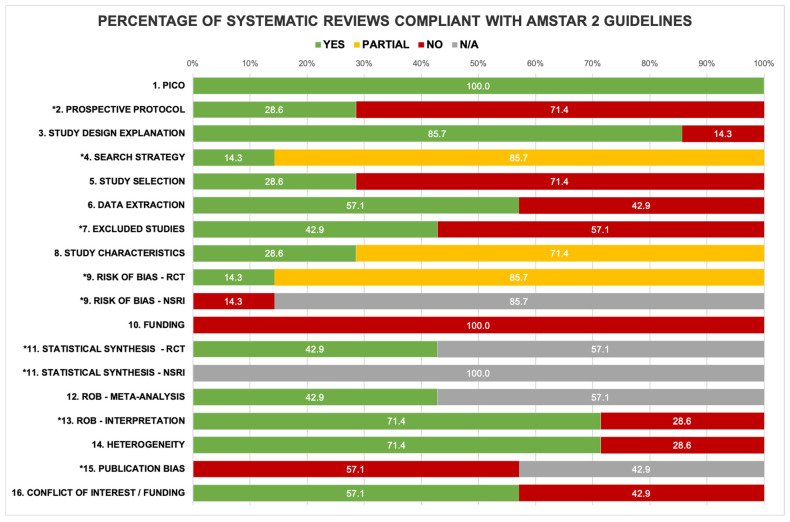
Percentage of systematic reviews compliant with AMSTAR 2 guidelines. * indicate critical domains.

The methodological quality of the included systematic reviews was assessed using the AMSTAR 2 [37]. The overall confidence in the results was rated as critically low for 86% (*n* = 6) of the systematic reviews and low for 14% (*n* = 1) [37]. Critical domains were marked with an asterisk in Figure 3 [37]. When systematic reviews did not perform meta-analysis [17,18,19,23], item 11 was scored “Not Applicable” and was not considered a critical domain [37]. In interpreting the results of the AMSTAR 2, four key points need to be mentioned. Only item 1—PICO question components—was completely fulfilled across systematic reviews, while item 10—reporting sources of funding of included studies—was not fulfilled by any systematic reviews. Of the critical domains, item 13—accounting for risk of bias in the interpretation of findings—demonstrated the highest percentage of systematic review compliance (71.4%, *n* = 5), and item 15—investigation of publication bias—demonstrated the lowest percentage of compliance (0%, *n* = 0). The complete analyses of the AMSTAR 2 are presented in Figure 3 and Table S4.

### 3.4. Reporting Quality

**Figure 4:**
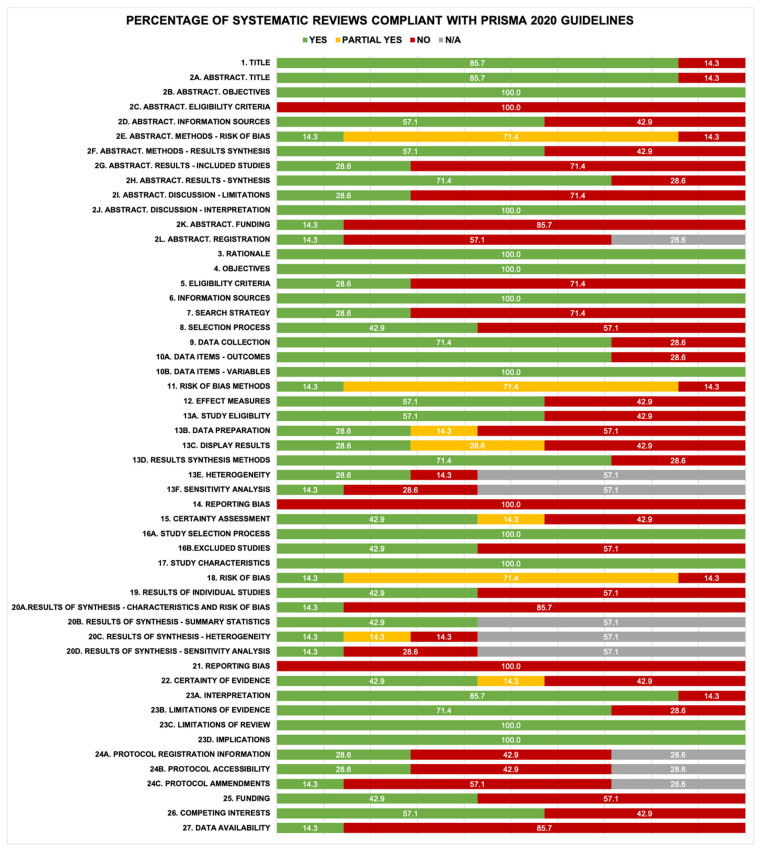
Percentage of systematic reviews compliant with PRISMA 2020 guidelines.

The reporting quality of the included systematic reviews was assessed using the PRISMA 2020 guidelines [38]. All systematic reviews were fully compliant (100%) with the ten reporting criteria: rationale and objectives in both the abstract and manuscript, providing a general interpretation of results in the abstract, information sources, defining variables (data items) for which data were sought, study selection process, study characteristics, limitations of the review, and implications of the results for practice. The guidelines with the lowest percentage of compliance (0%) were the complete reporting of eligibility criteria (inclusion and exclusion) in the abstract, and both the description of methodology and presentation of results regarding the assessment of risk of bias due to missing results in a synthesis (reporting bias). The complete analyses of the PRISMA guidelines are presented in Figure 4 and Table S5.

### 3.5. Pertinent Findings of Included Systematic Reviews

One systematic review with low risk of bias that conducted separate meta-analyses for each comparison intervention to TSM [20] demonstrated that TSM was more beneficial than thoracic mobilization, cervical mobilization, and standard care, but no better than cervical spine manipulation or placebo. Two systematic reviews ranging from low [21] to high risk of bias [22] performed meta-analysis across all included studies regardless of comparison intervention. Tsegay et al. [21] concluded that TSM alone or in combination with other treatments has immediate and short-term effects in individuals with chronic mechanical neck pain. Walser et al. [22] also demonstrated the short-term effects of TSM in individuals with neck pain for less than three months. The remaining four systematic reviews [17,18,19,23] (low to high risk of bias) in this umbrella review did not perform meta-analysis. Overall, these reviews highlighted variability in the quality of evidence that supports TSM as an effective short-term intervention in the management of individuals with neck pain. The conclusions of individual systematic reviews can be found in Table 1, with their risk of bias located in Table S3.

## 4. Discussion

Seven systematic reviews [17,18,19,20,21,22,23] that assessed the effectiveness of TSM alone and in combination with other manual therapy, exercise, modality, and education interventions were included in this manuscript with the overall risk of bias ranging from low to high. While all the included systematic reviews suggested that TSM is a viable short-term option for individuals with neck pain, the overall confidence in these results based on the AMSTAR 2 [37] ranged from low to critically low. The reporting quality was highly variable across PRISMA 2020 criteria, with compliance ranging from 0% to 100%.

Appraising the methodological rigor of the systematic reviews on TSM provides valuable insight for clinicians, policymakers, and payers when making evidence-based decisions regarding its role in patient management and healthcare resource allocation. Given the substantial overlap in primary studies included across systematic reviews, the findings regarding the short-term effectiveness of TSM were generally consistent. However, the confidence in these findings is limited as most reviews demonstrated critically low methodological quality and five did not perform meta-analysis. These limitations highlight the need for more robust and rigorous methodological systematic reviews. Although 71% of the included reviews were completed prior to the publication of the AMSTAR 2, none predated the original 2007 guidelines. This raises questions about whether the evidence guiding clinical decision making is misaligned with current research standards. Furthermore, only one review [20] implemented the appropriate tool to assess the risk of bias of RCTs (item 9). The remaining included reviews used the PEDro scale [17,18,19,21,22,23]; however, this instrument is not specifically designed to assess the risk of bias and rather assesses methodological quality. All future systematic reviews should perform a true risk of bias assessment for intended outcomes and assess the potential impact on the results of syntheses for a more rigorous evaluation of the existing literature.

The strength of the evidence is also called into question by inconsistent adherence to the PRISMA 2020 guidelines, suggesting a lack of transparency in reporting. Common deficiencies included the inadequate exploration of sources of heterogeneity, insufficient reporting of participant characteristics, and incomplete assessment of the risk of bias of the contributing studies. In addition, several key elements recommended for abstract reporting, such as clearly defined exclusion criteria, an adequate description of synthesis design and results, acknowledgment of the limitations of the review, and disclosure of funding sources, were often missing. However, this may reflect decisions in the prioritization of reported information given the editorial space limitations.

Among the seven reviews evaluated, only two [20,21] reported the development of a prospective or pre-specified protocol. This element is considered a critical domain on the AMSTAR 2, a required component of both the PRISMA 2020 abstract and main reporting guidelines, and a component of Domains 1 and 4 on the ROBIS tool. The development and availability of a pre-specified protocol is essential to enhance transparency, prevent selective reporting, and reduce bias by ensuring that objectives, inclusion criteria, and methods of data analysis are determined prospectively. In its absence, confidence in the methodological rigor and credibility of the evidence synthesis is significantly diminished. Similarly, inappropriate or inadequate search strategies led to downgraded methodological quality and increased risk for bias. According to the AMSTAR 2 guidelines, six reviews [17,18,19,21,22,23] did not implement a comprehensive literature search strategy, omitting key components such as searches of gray literature or trial registries and consultation with field experts. The ROBIS tool also revealed high concerns related to the potential omission of eligible studies in five of the reviews [17,18,21,22,23] attributed to factors such as inappropriate restrictions on language or publication date, insufficient reporting of the full search strategy and screening process, failure to search gray literature or trial registries, and omission of key databases. This introduces an increased risk for publication and selection bias and may undermine the reliability of the reviews’ findings.

The findings of this umbrella review contrast with a recent umbrella review by Reynolds et al. [36], which reported higher confidence in the systematic reviews of manual therapy for non-specific neck pain [36]. Only two systematic reviews included in Reynolds et al. [36] overlapped with the present umbrella review—Masaracchio et al. [20] and Tsegay et al. [21]—likely due to Reynolds et al.’s [36] eligibility criteria, which limited inclusion to reviews published between January 2016 and May 2023 due to the publication of the Clinical Practice Guidelines for neck pain in 2017 [16]. Notably, this restriction is inconsistent with ROBIS guidance and may have excluded relevant systematic reviews included in the present study. Furthermore, there is a notable discrepancy in methodological quality ratings, with Reynolds et al. [36] concluding moderate confidence in the evidence for these two reviews based on the AMSTAR 2, whereas the present review concluded critically low. Together, these findings highlight the need for further research to determine the optimal strategies for integrating manual therapy with other rehabilitation interventions within a multimodal plan of care [66,67,68]. Clinicians should base clinical decision making for individuals with mechanical neck pain on the totality of the available evidence. In the absence of contraindications, TSM should be strongly considered as part of the management approach, given its demonstrated short-term benefits and its potential to influence neurophysiological mechanisms [15] that may enhance outcomes when integrated with other therapeutic interventions. Finally, the limitations of the data hinder the ability to draw more robust conclusions regarding the effectiveness of TSM. Meta-meta-analysis was not feasible due to overlaps in the studies and the heterogeneity in synthesis methods [39]. Since the primary purpose was to perform an umbrella review, the authors did not perform a literature search designed to identify new primary studies to perform a new meta-analysis. According to the ROBIS guidance [47], this would not have been considered best practice and may have led to the exclusion of relevant RCTs that could have an important impact on the overall effect estimate. Therefore, this represents a major limitation of the existing evidence as strong conclusions based on sound quantitative analyses were not possible, leaving most findings presented narratively when comparing interventions. The exclusion of EMBASE and other databases may have resulted in the omission of relevant systematic reviews, potentially limiting the comprehensiveness of the evidence. Additionally, restricting eligibility to English language publications introduces the risk of language bias, as systematic reviews published in other languages may differ in methodological quality or reported findings. Together, these decisions may reduce the generalizability of the synthesized evidence and potentially influence the robustness of the conclusions. The discrepancies in quality ratings for Masaracchio et al. [20] and Tsegay et al. [21] compared with Reynolds et al. [36] suggest that subjective judgment and human error can influence quality appraisals, highlighting the importance of standardized evaluation processes to ensure reliable evidence synthesis.

## 5. Conclusions

While the overall confidence in the results of this umbrella review ranged from low to critically low, clinicians may consider implementing TSM as a potential short-term intervention for individuals with neck pain. Since firm conclusions about the true benefit of TSM cannot be drawn from this umbrella review, clinicians should consider the overall body of evidence, their clinical experience, and patient expectations as part of their clinical decision making. Future systematic reviews must develop and register prospective protocols, minimize risk of bias, and ensure compliance with established methodological and reporting guidelines to enhance the quality, transparency, and reproducibility of the evidence synthesis.

## Figures and Tables

**Table 1:** Description of studies.

Study *	Study Characteristics	Experimental Intervention	Comparison Intervention	Risk of Bias/Methodological Quality	Outcome Measures	Main Conclusions
Brown et al. 2014, [17] USA Systematic Review	13 studies ** (7 RCTs and 1 secondary analysis on TSM) *n* = 444 Population: Mechanical neck pain Age (range): 21–62 years Sex: NR Symptom duration (range): 9–1188 days	TSM: Supine, seated, or prone across included studies	CSM Cervical/thoracic mobilization Electro-thermal therapy Infrared laser and patient education Kinesiotape Rest No treatment	PEDro (range 5–9)	Pain Disability Cervical ROM	There was limited high-quality evidence comparing CSM and TSM. CSM and TSM have been shown to be equally valuable in relieving pain, disability, and improving ROM in patients with mechanical neck pain.
Cross et al. 2011, [18] USA Systematic Review	6 RCT *n* = 358 Population: Mechanical neck pain Age: NR Sex: NR Symptom duration (average): 3 months or less	TSM: Supine or seated across included studies	Cervical mobilization/strengthening Thoracic/cervical mobility exercise Placebo Heat/TENS Rest	PEDro (range 6–7)	Pain Disability Cervical ROM Adverse events	Despite weak evidence, TSM may provide short-term improvement in patients with acute or subacute mechanical neck pain. There were no statistically significant differences in adverse events.
Huisman et al. 2013, [19] The Netherlands Systematic Review	10 RCTs *n* = 677 Population: Non-specific neck pain Age: NR Sex: NR Symptom duration: Acute, subacute, and chronic	TSM: Supine or seated, but generally poorly described by included studies + exercise, education, infrared radiation	CSM Thoracic mobilization Placebo TSM Exercise Education	PEDro (range 4–8)	Pain Disability	TSM has therapeutic benefits to some patients with neck pain. However, there is insufficient evidence that TSM is more effective than control interventions or CSM in reducing pain and disability in patients with non-specific neck pain.
Masaracchio et al. 2019, [20] USA Systematic Review and Meta-Analysis	14 RCTs *n* = 885 Population: Mechanical neck pain Age (average range): 32.5–46.84 years Sex: 61.7% female, 38.3% male Symptom duration: Acute, subacute, and chronic	TSM: Supine, seated, standing, or prone across included studies + exercise, cervical mobilization, infrared therapy, education	CSM Cervical/thoracic mobilization Placebo TSM Modalities Exercise Standard care Education	RoB (range 7–12)	Pain Disability GROC Adverse events	TSM is more beneficial, without any adverse events and minimal unwanted side effects, than thoracic mobilization, cervical mobilization, and standard care, but no better than CSM or placebo TSM to improve pain and disability for the management of individuals with mechanical neck pain.
Tsegay et al. 2023, [21] Ethiopia Systematic Review and Meta-Analysis	8 RCTs *n* = 385 Population: Chronic mechanical neck pain Age (range): 18–60 years Sex: NR Symptom duration: Chronic	TSM: Supine or prone across included studies	CSM Thoracic spine mobilization Placebo TSM Infrared therapy DNF exercise/cervical stability training	PEDro (range 4–8)	Pain Disability	TSM alone or in combination with other treatment has produced an immediate and short-term effect to improve pain and neck disability among patients with chronic mechanical neck pain.
Walser et al. 2009, [22] USA Systematic Review and Meta-Analysis	13 RCTs *** (9 studies on neck pain) *n* = 372 Population: Neck pain Age (average range): 25–48 years Sex: 159 M, 213 F Symptom duration: 12 days to 3 months	TSM: Most commonly supine anterior-to-posterior across included studies	Not specified	PEDro (Range 4–9)	Pain Disability	There is currently sufficient evidence to support the use of TSM for the management of neck conditions in specific subgroups of patients for short-term outcomes.
Young et al. 2013, [23] USA Systematic Review	14 studies (10 RCTs, 1 quasi-experimental, 1 prospective cohort, 1 case series, 1 secondary analysis) *n* = 805 Population: Mechanical neck pain Age: 18–60 years Sex: NR Symptom duration: NR	TSM: Supine or seated across included studies + modalities	CSM Thoracic mobilization Placebo TSM Modalities Exercise Education	PEDro (range 3–9)	Pain Disability Cervical ROM	A significant amount of evidence, although of varied quality, exists for the short-term benefits of TSM in patients with mechanical neck pain.

* Studies are listed alphabetically. ** Brown et al. 2014 [17] included articles assessing the effects of both TSM and CSM. Data on TSM as the experimental intervention were isolated by removing articles with CSM only or combined CSM and TSM from the study count and sample size. *** Walser et al. 2009 [22] included articles assessing the effects of TSM on patients with musculoskeletal conditions. Data on TSM for patients with neck pain were isolated by removing articles on shoulder and trunk pain from the study count and sample size. Abbreviations: CSM, cervical spine manipulation; F, female; GROC, global rating of change scale; M, male; NR, Not reported; ROM, range of motion; PEDro, Physiotherapy Evidence Database scale; RCT, randomized controlled trial; RoB, risk of bias tool; TSM, thoracic spine manipulation; TENS, transcutaneous electrical stimulation; USA, United States of America.

## Data Availability

No new data were created or analyzed in this study.

## Supplementary Materials

The following supporting information can be downloaded at https://www.mdpi.com/article/10.3390/healthcare14020240/s1, Table S1: Search strategy; Table S2: Excluded studies; Table S3: ROBIS assessment data of included systematic reviews; Table S4: AMSTAR 2 criteria data of included systematic reviews; Table S5: PRISMA 2020 guideline data of included systematic reviews.

## Abbreviations

The following abbreviations are used in this manuscript:

TSM	Thoracic Spine Manipulation
RI	Regional Interdependence
AMSTAR 2	A Measurement Tool to Assess Systematic Reviews 2
PRISMA	Preferred Reporting Items for Systematic Reviews and Meta-Analyses
RCT	Randomized Controlled Trial
PEDro	Physiotherapy Evidence Database
ROBIS	Risk of Bias in Systematic Reviews
GRADE	Grading of Recommendations Assessment, Development, and Evaluation
CCA	Corrected Covered Area
