# Supplementary material for: Effectiveness of Thoracic Spine Manipulation for the Management of Neck Pain: A Systematic Umbrella Review with Risk of Bias and Methodological and Reporting Quality
Source: Healthcare (Basel). 2026 Jan 18;14(2):240. doi: 10.3390/healthcare14020240 (PMC12841215; doi:10.3390/healthcare14020240)
Supplement: Supplementary file 1 [file healthcare-14-00240-s001.zip › Table S1_Search Strategy.pdf]

Table S1. Search Strategy

**Database**	**Search Strategy**	**Yield**
PubMed	("Neck Pain"[Mesh] OR cervicalgia[tw] OR “mechanical neck pain”[tw] OR “neck pain”[tw] OR “cervical pain”[tw]) AND ("Manipulation, Spinal"[Mesh] OR high-velocity low amplitude[tw] OR HVLA[tw] OR “thrust manip*”[tw] OR “spine manip*”[tw] OR “spinal manipulative therapy”[tw] OR “thoracic manip*”[tw]) AND systematic[Filter]	46
CINAHL (EBSCO Host)	((MH "Neck Pain") OR cervicalgia OR "mechanical neck pain" OR "neck pain" OR "cervical pain") AND ((MH "Manipulation, Orthopedic") OR (MH “Manipulation, Chiropractic”) OR high-velocity low-amplitude OR HVLA OR "thrust manip*" OR "spine manip*" OR "spinal manipulative therapy" OR "thoracic manip*") AND (MH "Systematic Review")	44
Cochrane Library	((MeSH descriptor: [Neck Pain] explode all trees) OR “Mechanical neck pain” OR “Cervical pain” OR “Neck pain” OR “cervicalgia”) AND ((MeSH descriptor: [Musculoskeletal Manipulations] explode all trees) OR (MeSH descriptor: [Manipulation, Spinal] explode all trees) OR “Thoracic manipulation” OR “Thrust manipulation” OR “Spinal manipulation” OR high-velocity low-amplitude OR HVLA OR “spinal manipulative therapy)	20
Total		110
After duplicates removed		90
