# Supplementary material for: Effectiveness of Thoracic Spine Manipulation for the Management of Neck Pain: A Systematic Umbrella Review with Risk of Bias and Methodological and Reporting Quality
Source: Healthcare (Basel). 2026 Jan 18;14(2):240. doi: 10.3390/healthcare14020240 (PMC12841215; doi:10.3390/healthcare14020240)
Supplement: Supplementary file 1 [file healthcare-14-00240-s001.zip › Table S2_Excluded Studies.pdf]

Table S2. Excluded Studies

Reason for Exclusion	References
Outdated Cochrane Review<br>1 study	• Gross A, Miller J, D'Sylva J, et al. Manipulation or mobilisation for neck pain. *Cochrane Database Syst Rev.* 2010(1):Cd004249.
Wrong intervention (thoracic spine manipulation could not be identified as the experimental intervention)<br>10 studies	• Carrasco-Uribarren A, Pardos-Aguilella P, Jiménez-Del-Barrio S, Cabanillas-Barea S, Pérez-Guillén S, Ceballos-Laita L. Cervical manipulation versus thoracic or cervicothoracic manipulations for the management of neck pain. A systematic review and meta-analysis. *Musculoskelet Sci Pract.* 2024;71:102927.<br><br>• Chaibi A, Stavem K, Russell MB. Spinal Manipulative Therapy for Acute Neck Pain: A Systematic Review and Meta-Analysis of Randomised Controlled Trials. *J Clin Med.* 2021;10(21).<br><br>• Guevara Hernández DM, Ortiz Perez SM, Baltodano Ardón F, Ocaña Villacrés YD, Tello Nunez CL, Pineda Grillo IJ. Effectiveness of spinal manipulation on chronic cervical pain: a systematic review of randomized clinical trials. *Salud, Ciencia y Tecnología.* 2024;4(1):1-8.<br><br>• Hidalgo B, Hall T, Bossert J, Dugeny A, Cagnie B, Pitance L. The efficacy of manual therapy and exercise for treating non-specific neck pain: A systematic review. *Journal of Back & Musculoskeletal Rehabilitation.* 2017;30(6):1149-1169.<br><br>• Hurwitz EL, Aker PD, Adams AH, Meeker WC, Shekelle PG. Manipulation and mobilization of the cervical spine. A systematic review of the literature. *Spine (Phila Pa 1976).* 1996;21(15):1746-1759; discussion 1759-1760.<br><br>• Minnucci S, Innocenti T, Salvioli S, et al. Benefits and Harms of Spinal Manipulative Therapy for Treating Recent and Persistent Nonspecific Neck Pain: A Systematic Review With Meta-analysis. *J Orthop Sports Phys Ther.* 2023;53(9):510-528.<br><br>• Schroeder J, Kaplan L, Fischer DJ, Skelly AC. The outcomes of manipulation or mobilization therapy compared with physical therapy or exercise for neck pain: A systematic review. *Evid Based Spine Care J.* 2013;4(1):30-41. doi:10.1055/s-0033-1341605<br><br>• Vernon HT, Humphreys BK, Hagino CA. A systematic review of conservative treatments for acute neck pain not due to whiplash. *J Manipulative Physiol Ther.* 2005;28(6):443-448.<br><br>• Vincent K, Maigne JY, Fischhoff C, Lanlo O, Dagenais S. Systematic review of manual therapies for nonspecific neck pain. *Joint Bone Spine.* 2013;80(5):508-515.

	• Yang J, Zhao S, Zhang R, et al. Effectiveness and safety of thoracic manipulation in the treatment of neck pain: An updated systematic review and meta-analysis. *Technol Health Care.* 2024;32(S1):385-402.
Wrong population (findings were not specific to individuals with mechanical neck pain)<br>4 studies	• Coronado RA, Bialosky JE, Cook CE. The temporal effects of a single session of high-velocity, low-amplitude thrust manipulation on subjects with spinal pain. *Physical Therapy Reviews.* 2010;15(1):29-35.<br><br>• Diao Y, Liu Y, Pan J, et al. Efficacy and safety of spinal manipulative therapy in the management of acute neck pain: a systematic review and meta-analysis. *Syst Rev.* 2025;14(1):97.<br><br>• Gross AR, Hoving JL, Haines TA, et al. A Cochrane review of manipulation and mobilization for mechanical neck disorders. *Spine (Phila Pa 1976).* 2004;29(14):1541-1548.<br><br>• Gross A, Langevin P, Burnie SJ, et al. Manipulation and mobilisation for neck pain contrasted against an inactive control or another active treatment. *Cochrane Database Syst Rev.* 2015;2015(9):Cd004249.
Wrong publication type<br>1 study	• Shekelle PG, Paige NM, Miake-Lye IM, Beroes JM, Booth MS, Shanman R. VA Evidence-based Synthesis Program Reports. In: *The Effectiveness and Harms of Spinal Manipulative Therapy for the Treatment of Acute Neck and Lower Back Pain: A Systematic Review.* Washington (DC): Department of Veterans Affairs (US); 2017.
Wrong study design<br>1 study	• Bronfort G, Haas M, Evans RL, Bouter LM. Efficacy of spinal manipulation and mobilization for low back pain and neck pain: a systematic review and best evidence synthesis. *Spine J.* 2004;4(3):335-356.
