# Supplementary material for: Effectiveness of Thoracic Spine Manipulation for the Management of Neck Pain: A Systematic Umbrella Review with Risk of Bias and Methodological and Reporting Quality
Source: Healthcare (Basel). 2026 Jan 18;14(2):240. doi: 10.3390/healthcare14020240 (PMC12841215; doi:10.3390/healthcare14020240)
Supplement: Supplementary file 1 [file healthcare-14-00240-s001.zip › Table S3_ROBIS Data.pdf]

Table S3. ROBIS assessment data of included systematic reviews [47]

Phase 1: Assessing relevance

Study	Relevance
Brown 2014	Yes
Cross 2011	Yes
Huisman 2013	Yes
Masaracchio 2019	Yes
Tsegay 2023	Yes
Walser 2009	Yes
Young 2013	Yes
For each study, the relevance to the target question was judged as “Yes,” “No,” “Partial,” or “Unclear” based on the criteria published by Whiting et al. [47]	

Phase 2: Identifying concerns with the review process

	Domain 1: Study Eligibility						Domain 2: Identification and Selection of Studies						Domain 3: Data Collection and Study Appraisal						Domain 4: Synthesis and Findings						
**Study**	**1.1**	**1.2**	**1.3**	**1.4**	**1.5**	**Concerns**	**2.1**	**2.2**	**2.3**	**2.4**	**2.5**	**Concerns**	**3.1**	**3.2**	**3.3**	**3.4**	**3.5**	**Concerns**	**4.1**	**4.2**	**4.3**	**4.4**	**4.5**	**4.6**	**Concerns**
Brown 2014	NI	Y	N	Y	Y	High	N	N	PN	N	NI	High	PN	Y	NA	Y	Y	Low	NA	NI	N	NI	NA	N	Unclear
Cross 2011	NI	Y	N	Y	Y	High	N	Y	PN	N	Y	High	PY	Y	Y	Y	Y	Low	NA	NI	Y	Y	NA	N	Unclear
Huisman 2013	NI	Y	N	Y	Y	Low	Y	Y	PY	Y	NI	Low	NI	Y	NA	Y	Y	Low	NA	NI	Y	Y	NA	Y	Low
Masaracchio 2019	Y	Y	N	Y	PN	Low	Y	Y	Y	PN	Y	Low	PN	Y	Y	Y	Y	Low	Y	Y	Y	Y	N	Y	Low
Tsegay 2023	Y	Y	N	N	N	High	N	Y	N	N	Y	High	PY	Y	Y	Y	Y	Low	Y	Y	N	N	N	Y	High
Walser 2009	NI	N	N	PY	PN	High	N	Y	PN	I	N	High	NI	N	Y	Y	N	High	Y	NI	PY	N	N	N	High
Young 2013	NI	N	N	N	PN	High	N	N	N	Y	NI	High	PN	Y	NA	Y	Y	Low	NA	NI	N	NI	NA	Y	Unclear

For each signaling question, “Y; Yes” was assigned if the criteria was fulfilled; “PY; Probably Yes” was assigned if the criteria was probably fulfilled given the information provided; “N; No” was assigned if the criteria was not fulfilled; “PN; Probably No” was assigned if the criteria was probably not fulfilled given the information provided; “NI; No information” was assigned if there is insufficient data reported to permit a judgment. For select criteria, “NA; Not applicable” was assigned if there was no synthesis of study results to allow judgment of the criteria.

For each domain, “Low,” “High,” or “Unclear” level of concern was judged based on the criteria published by Whiting et al. [47]

Phase 3: Judging risk of bias

	Concern				Risk of Bias in the Review	Rationale
**Study**	**Domain 1**	**Domain 2**	**Domain 3**	**Domain 4**		
Brown 2014	High	High	Low	Unclear	High	The interpretation of findings addressed concerns from domain 1 related to the eligibility criteria, but did not address concerns from domain 2 related to the search strategy or domain 4 related to the synthesis and findings. All studies in the review were not directly applicable to the review's research question and no formal quality assessment was conducted, however, the relevance of the included studies was reflected in the discussion. A balanced account of all analyses was presented.
Cross 2011	High	High	Low	Unclear	High	The interpretation of findings addressed some concerns from domain 1 related to the eligibility criteria and domain 2 related to the databases searched, but did not address concerns from domain 1 related to a prespecified protocol, domain 2 related to the availability of a full search strategy or domain 4 related to the synthesis and findings. All

						studies in the review were directly applicable to the review's research question. A balanced account of all analyses was presented.
Huisman 2013	Low	Low	Low	Low	Low	All domains assessed during phase 2 were rated as low concerns, therefore, the findings of this review are likely to be reliable. The conclusions were supported by evidence and included consideration of the relevance of included studies. All studies in the review were directly applicable to the review's research question. A balanced account of all analyses was presented
Masaracchio 2019	Low	Low	Low	Low	Low	All domains assessed during phase 2 were rated as low concerns, therefore, the findings of this review are likely to be reliable. The conclusions were supported by evidence and included consideration of the relevance of included studies. All studies in the review were directly applicable to the review's research question. A balanced account of all analyses was presented.
Tsegay 2023	High	High	Low	High	Low	The interpretation of findings addressed concerns from all domains, acknowledging potential limitations and the impact on findings. All studies in the review were directly applicable to the review's research question. A balanced account of all analyses was presented.
Walser 2009	High	High	High	High	High	The interpretation of findings addressed some concerns from domain 1 related to the eligibility criteria restricting studies not published in the English language, and domain 3 related to performance of methodological quality assessment by only one reviewer. However, additional concerns from domains 1, 2, 3, and 4 were not addressed. All studies in the review were directly applicable to the review's research question. A balanced account of all analyses was presented.
Young 2013	High	High	Low	Unclear	High	The interpretation of findings addressed some concerns from domain 1 related to the eligibility criteria restricting studies not published in the English language, but did not address additional concerns from domain 1 or concerns from domains 2 or 4. All studies in the review were not directly applicable to the review's research question, however, the relevance of the included studies was reflected in the GRADE analysis and reflected in the discussion. A balanced account of all analyses was presented.
An overall judgment of risk of bias was judged as “Low,” “High,” or “Unclear” based on the criteria published by Whiting et al. [47]						
