# Supplementary material for: Effectiveness of Thoracic Spine Manipulation for the Management of Neck Pain: A Systematic Umbrella Review with Risk of Bias and Methodological and Reporting Quality
Source: Healthcare (Basel). 2026 Jan 18;14(2):240. doi: 10.3390/healthcare14020240 (PMC12841215; doi:10.3390/healthcare14020240)
Supplement: Supplementary file 1 [file healthcare-14-00240-s001.zip › Table S4_AMSTAR 2 Data.pdf]

**Table S4.** AMSTAR 2 criteria data of included systematic reviews [37]

| **Study** | **1** | ***2** | **3** | ***4** | **5** | **6** | ***7** | **8** | ***9 RCT** | ***9 NSRI** | **10** | ***11 RCT** | ***11 NSRI** | **12** | ***13** | **14** | ***15** | **16** | **Critical Domain Weakness** | **Non-critical Domain Weakness** | **Overall Confidence** |
|---|---|---|---|---|---|---|---|---|---|---|---|---|---|---|---|---|---|---|---|---|---|
| Brown 2014 | Y | N | Y | P | N | Y | N | P | P | NA | N | NA | NA | NA | N | Y | NA | Y | 3** | 2 | Critically Low |
| Cross 2011 | Y | N | N | P | Y | Y | Y | P | P | NA | N | NA | NA | NA | N | Y | NA | N | 2** | 3 | Critically Low |
| Huisman 2013 | Y | N | Y | P | N | N | Y | P | P | NA | N | NA | NA | NA | Y | Y | NA | Y | 1** | 3 | Low |
| Masaracchio 2019 | Y | Y | Y | Y | Y | N | N | Y | Y | NA | N | Y | NA | Y | Y | Y | N | Y | 2 | 2 | Critically Low |
| Tsegay 2023 | Y | Y | Y | P | N | Y | N | Y | P | NA | N | Y | NA | Y | Y | Y | N | Y | 2 | 2 | Critically Low |
| Walser 2009 | Y | N | Y | P | N | N | Y | P | P | NA | N | Y | NA | Y | Y | N | N | N | 2 | 5 | Critically Low |
| Young 2013 | Y | N | Y | P | N | Y | N | P | P | N | N | NA | NA | NA | Y | N | N | N | 3** | 5 | Critically Low |
| Compliance Percentage | 100 | 28.6 | 85.7 | 14.3 | 28.6 | 57.1 | 42.9 | 28.6 | 14.3 | 0 | 0 | 42.9 | 0 | 42.9 | 71.4 | 71.4 | 0 | 57.1 | | | |

For each item, “Y; Yes” or “P; Partial Yes” was awarded and the applicable AMSTAR 2 criteria were selected depending on the level of adherence to the domain criteria, and “N; No” was assigned if there was no information related to the domain or if the presented information could not justify partial or full adherence to the criteria. For select criteria, “Not applicable” was chosen depending on the design of the systematic review as it applied to the AMSTAR 2 criteria.
*Critical domain
**For systematic reviews that did not perform meta-analysis, item 11 was not considered a critical domain.
