# Supplementary material for: Effectiveness of Thoracic Spine Manipulation for the Management of Neck Pain: A Systematic Umbrella Review with Risk of Bias and Methodological and Reporting Quality
Source: Healthcare (Basel). 2026 Jan 18;14(2):240. doi: 10.3390/healthcare14020240 (PMC12841215; doi:10.3390/healthcare14020240)
Supplement: Supplementary file 1 [file healthcare-14-00240-s001.zip › Table S5_PRISMA data.pdf]

Table S5. PRISMA 2020 guideline data of included systematic reviews [38]

**Study**	**1**	**3**	**4**	**5**	**6**	**7**	**8**	**9**	**10a**	**10b**	**11**	**12**	**13a**	**13b**	**13c**	**13d**	**13e**	**13f**	**14**	**15**
Brown 2014	N	Y	Y	N	Y	N	N	Y	Y	Y	P	N	Y	N	N	N	NA	NA	N	N
Cross 2011	Y	Y	Y	N	Y	N	Y	Y	Y	Y	P	Y	Y	P	N	Y	NA	NA	N	N
Huisman 2013	Y	Y	Y	N	Y	N	N	N	N	Y	P	N	N	N	Y	Y	NA	NA	N	P
Masaracchio 2019	Y	Y	Y	Y	Y	Y	Y	Y	Y	Y	Y	Y	Y	Y	Y	Y	Y	Y	N	Y
Tsegay 2023	Y	Y	Y	N	Y	Y	Y	Y	Y	Y	P	Y	N	N	P	Y	N	N	N	Y
Walser 2009	Y	Y	Y	Y	Y	N	N	N	Y	Y	N	Y	Y	Y	P	Y	Y	N	N	N
Young 2013	Y	Y	Y	N	Y	N	N	Y	N	Y	P	N	N	N	N	N	NA	NA	N	Y
Compliance Percentage	85.7	100	100	28.6	100	28.6	42.9	71.4	71.4	100	14.3	57.1	57.1	28.6	28.6	71.4	28.6	14.3	0	42.9

(continued)

**Study**	**16a**	**16b**	**17**	**18**	**19**	**20a**	**20b**	**20c**	**20d**	**21**	**22**	**23a**	**23b**	**23c**	**23d**	**24a**	**24b**	**24c**	**25**	**26**	**27**
Brown 2014	Y	N	Y	P	N	N	NA	NA	NA	N	N	Y	Y	Y	Y	N	N	N	Y	Y	N
Cross 2011	Y	Y	Y	P	Y	N	NA	NA	NA	N	N	Y	Y	Y	Y	NA	NA	NA	N	N	N
Huisman 2013	Y	Y	Y	P	N	Y	NA	NA	NA	N	P	Y	Y	Y	Y	N	N	N	Y	Y	N
Masaracchio 2019	Y	N	Y	Y	Y	N	Y	P	Y	N	Y	Y	Y	Y	Y	Y	Y	Y	Y	Y	Y
Tsegay 2023	Y	N	Y	P	Y	N	Y	N	N	N	Y	Y	N	Y	Y	Y	Y	N	N	Y	N
Walser 2009	Y	Y	Y	N	N	N	Y	Y	N	N	N	Y	N	Y	Y	NA	NA	NA	N	N	N
Young 2013	Y	N	Y	P	N	N	NA	NA	NA	N	Y	N	Y	Y	Y	N	N	N	N	N	N
Compliance Percentage	100	42.9	100	14.3	42.9	14.3	42.9	14.3	14.3	0	42.9	85.7	71.4	100	100	28.6	28.6	14.3	42.9	57.1	14.3

For each item, “Y; Yes” was awarded if the information was included in the publication, “N; No” was assigned if the information was unclear, incomplete, or not included, “NA, Not applicable” was assigned if the criteria did not apply to the systematic review, and “P; Partial Yes” was considered if the information was reported in the manuscript, but was located in the wrong section according to the PRISMA 2020 guidelines or when systematic reviews used the PEDro scale to assess methodological quality rather than a tool designed to assess risk of bias.

PRISMA 2020 guideline abstract data of included systematic reviews

Study	2a	2b	2c	2d	2e	2f	2g	2h	2i	2j	2k	2l
Brown 2014	N	Y	N	Y	P	N	N	N	N	Y	N	N
Cross 2011	Y	Y	N	N	N	Y	N	Y	Y	Y	N	NA
Huisman 2013	Y	Y	N	Y	P	Y	Y	Y	N	Y	Y	N
Masaracchio 2019	Y	Y	N	Y	Y	Y	N	Y	Y	Y	Y	Y
Tsegay 2023	Y	Y	N	Y	P	Y	Y	Y	N	Y	N	N
Walser 2009	Y	Y	N	N	P	N	N	Y	N	Y	N	NA
Young 2013	Y	Y	N	N	P	N	N	N	N	Y	N	N
Compliance Percentage	85.7	100	0	57.1	14.3	57.1	28.6	71.4	28.6	100	14.3	14.3
For each item, “Y; Yes” was awarded if the information was included in the publication, “N; No” was assigned if the information was unclear, incomplete, or not included, and “NA, Not applicable” was assigned if the criteria did not apply to the systematic review.												
